# Fish Oil Enhances Recovery of Intestinal Microbiota and Epithelial Integrity in Chronic Rejection of Intestinal Transplant

**DOI:** 10.1371/journal.pone.0020460

**Published:** 2011-06-17

**Authors:** Qiurong Li, Qiang Zhang, Chenyang Wang, Chun Tang, Yanmei Zhang, Ning Li, Jieshou Li

**Affiliations:** Department of Surgery, Jinling Hospital, Nanjing University School of Medicine, Nanjing, China; The University of Kansas Medical Center, United States of America

## Abstract

**Background:**

The intestinal chronic rejection (CR) is the major limitation to long-term survival of transplanted organs. This study aimed to investigate the interaction between intestinal microbiota and epithelial integrity in chronic rejection of intestinal transplantation, and to find out whether fish oil enhances recovery of intestinal microbiota and epithelial integrity.

**Methods/Principal Findings:**

The luminal and mucosal microbiota composition of CR rats were characterized by DGGE analysis at 190 days after intestinal transplant. The specific bacterial species were determined by sequence analysis. Furthermore, changes in the localization of intestinal TJ proteins were examined by immunofluorescent staining. PCR-DGGE analysis revealed that gut microbiota in CR rats had a shift towards *Escherichia coli*, *Bacteroides spp* and *Clostridium spp* and a decrease in the abundance of *Lactobacillales* bacteria in the intestines. Fish oil supplementation could enhance the recovery of gut microbiota, showing a significant decrease of gut bacterial proportions of *E. coli* and *Bacteroides spp* and an increase of *Lactobacillales spp*. In addition, CR rats showed pronounced alteration of tight junction, depicted by marked changes in epithelial cell ultrastructure and redistribution of occuldin and claudins as well as disruption in TJ barrier function. Fish oil administration ameliorated disruption of epithelial integrity in CR, which was associated with an improvement of the mucosal structure leading to improved tight junctions.

**Conclusions/Significance:**

Our study have presented novel evidence that fish oil is involved in the maintenance of epithelial TJ integrity and recovery of gut microbiota, which may have therapeutic potential against CR in intestinal transplantation.

## Introduction

The intestinal chronic rejection (CR) is the major cause of late allograft dysfunction and it appears to be a significant problem in long-term survival of the patients [1], [2]. Pathological changes, including vasculopathy, parenchymal fibrosis and inflammatory cells infiltration in the graft, are the hallmarks of CR [3]. The application of new potent immunosuppressive drugs has made small bowel transplantation clinically feasible for an increasing number of patients [1], [4]. However, the chronic rejection remains major concerns [1], [2], [4]. The mechanisms underlying CR are far from clear.

The human gastrointestinal microbiota is a complex ecosystem of around 300–500 bacterial species and the number of bacteria reaches extremely high densities (10^11^ to 10^12^ cells/ml). The gut microbiota impacts upon a wide range of host biological processes and it plays an important role in the intestinal immune system, host metabolism, cell renewal in the intestinal epithelium, intestinal angiogenesis and motility [5], [6], and intestinal pathogens exclusion [7]. The structure of the gut microbiota has also been implicated in the etiology of a spectrum of intestinal diseases, including inflammatory bowel disease (IBD), colorectal cancer, infection and inflammation [8].

The gut is considered as the primary site for cross-talk between the host and microorganisms. The intestinal epithelium constitutes the major interface between the microbiota and internal host tissues. The gut microbiota directly interacts with the intestinal epithelium, involving in the development of the mucosal epithelium and maturation of the mucosal immune system [9] and it provides resistance to colonization by pathogenic microorganisms [10]. Normally, the intestinal epithelium serves as a continuous barrier to avoid bacteria translocation and some endogenous or exogenous events may alter intestinal barrier function and induce leaky gut. Tight junctions (TJs) are specialized plasma membrane and play an important role in barrier function. Proteins of tight junction are composed of occludin, the claudin family, junctional adhesion molecule-JAM and ZO-1, ZO-2 and ZO-3 [11], [12]. Our findings indicated the interaction between inflammatory cytokines and alterations of lipid composition in membrane microdomains of TJs in the inflammatory processes [13].

Relatively few studies have investigated the interaction between intestinal epithelium and the microbial communities during disease and homeostasis. Changes in gut microbiota control inflammation and improve gut permeability in obese mice [14]. In addition, we have demonstrated the *in vivo* functional role of Paneth cells in maintaining homeostasis of microbial community [15] and the changes of intestinal intraepithelial lymphocytes following alemtuzumab treatment [16]. It has recently been shown changes in gut microbiota and increase of intestinal permeability by reducing the expression of two tight junction proteins ZO-1 and occludin [14] in obese mice. Importantly, however, there is no direct evidence for the interaction between the altered gut microbiota and changes in the structure and function of tight junction.

n-3 polyunsaturated fatty acids (PUFAs), which are rich in fish oil (FO), have positive influence on inflammatory disorders, such as Alzheimer's disease, lung fibrosis, and inflammatory bowel disease [17]–[19], and play important roles in gut integrity and epithelial barrier function [20], [21]. The cytokine-induced permeability changes were alleviated through a mechanism involving the redistribution of occludin and ZO-1 by n-3 PUFAs [22]. However, the potential roles played by n-3 PUFAs in tight junction in chronic rejection of intestinal transplantation have not been investigated. The interactions of PUFAs with the indigenous microbiota might affect the biological roles of gut. In vitro studies on the effects of n-3 PUFAs on the growth and adhesion of different *Lactobacillus* strains have shown different results depending on the strain [23]. The administration of PUFAs has positively influenced the adhesion of *Lactobacillus* to the mucosa of gnotobiotic piglets [24]. But only few studies associated with the effects of n-3 PUFAs on gut microtiota were found.

In the transplanted intestine of chronic rejection, there are remarkable complex interactions in rejection and adaptation between the allograft and the host. Therefore, a specific composition of the microbial communities may develop and a shared host of the transplanted intestine may form in chronic rejection of intestinal transplantation. Up to date, the alteration of such microbial community structure in chronic rejection of intestinal transplant is unknown. Fish oil had been shown to inhibit CR in animal models of cardiac transplantation [25], [26]. Our findings show that fish oil decreases allograft inflammation and significantly attenuates the development of allograft CR in intestinal transplantation [27]. However, effects of n-3 PUFAs on microbial community structure in chronic rejection of intestinal transplant are poorly understood.

We hypothesize that the interaction between the altered microbiota composition and tight junction play a vital role in intestinal transplantation during chronic rejection. To confirm the hypothesis, we compared the differences in the gut microbiota composition between the syngeneic group and allogenic groups at 190 days after intestinal transplant. The luminal and mucosal microbiota composition of the intestinal transplant rats were characterized by DGGE analysis. Furthermore, we aimed to identify specific bacterial species of the microbiota that could be linked to intestinal transplantation by sequence analysis. And the effect of chronic rejection on the localization of intestinal tight junctions-associated proteins was examined by immunofluorescent staining. In addition, the beneficial effect of fish oil on tight junction structure and the recovery of the bacterial population structure were studied.

## Materials and Methods

### Small bowel transplantation and experimental groups

Orthotopic transplantation of the entire jejunum and ileum was performed as previously reported [28], using 200 to 300 g Male Fisher 344 (F344) and Lewis rats (Vitalriver Company, Beijing, China) as donors and recipients, respectively. The recipient's small intestine was resected and replaced with the donor's intestine. In the recipient, the graft superior mesenteric artery was anastomosed to the recipient's infrarenal aorta in an end-to-side manner, and the graft portal vein to recipient's infrarenal inferior vena cava. The intestinal continuity was restored with an end-to-end anastomosis. All experimental procedures were performed in accordance with the “Guide for the Care and Use of Laboratory Animals” published by the National Institutes of Health (NIH publication 86–23 revised 1985). And the protocols were approved by the Animal Research Committee of Nanjing University.

Syngeneic (n = 8) and allogeneic transplant groups (eight animals in each group) were involved in our study. The allogeneic rats were subdivided into the following three groups: PBS group (Allo/PBS), the rats were treated with phosphate buffered saline (PBS, 0.1 M, pH 7.4); CO group (Allo/CO), rats were fed with corn oil in a dosage of 6 ml/kg each day after intestinal transplantation; FO group (Allo/FO), rats received 6 ml/kg fish oil each day. FK506 was administered intramuscularly at a daily dosage of 1.0 mg/kg on postoperative days 0–13, 20, 27.

For the other detailed methods see Text S1.

## Results

### Enhancement of recovery in the gut microbiota in CR rats by long-term fish oil supplementation

**Figure 1:**
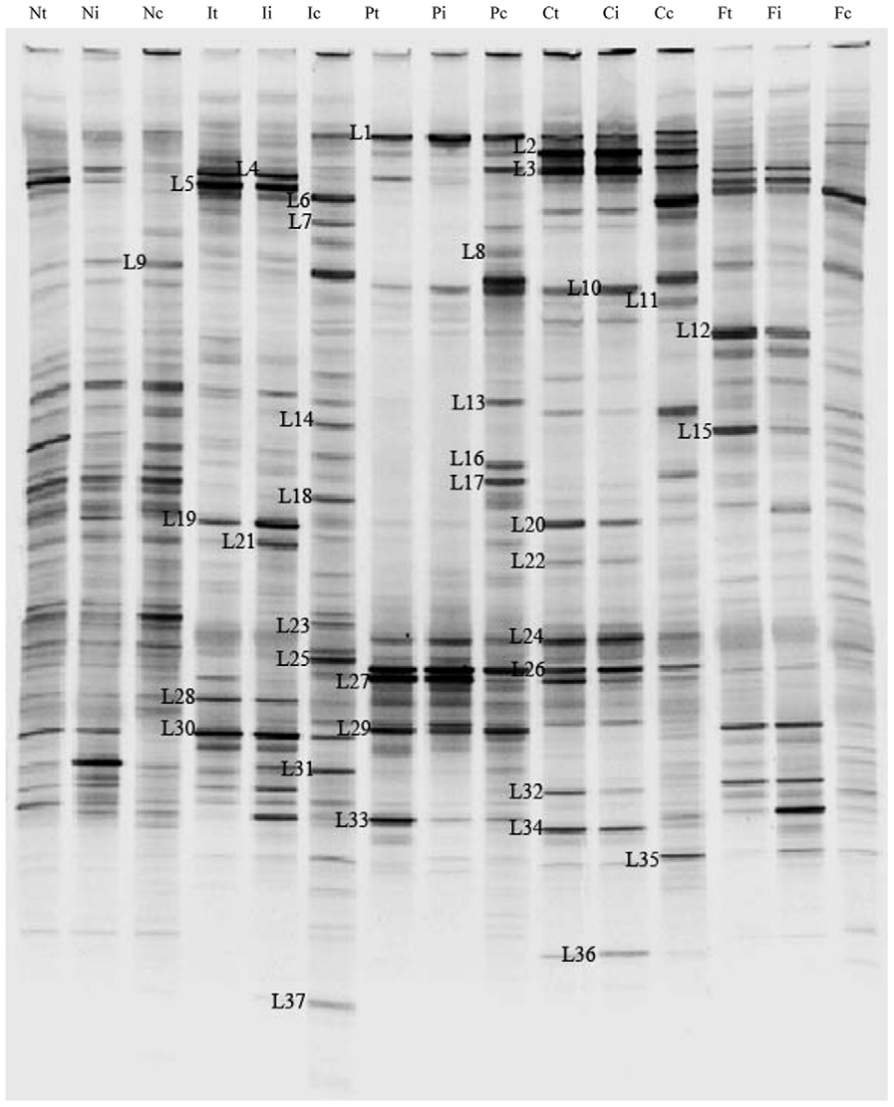
DGGE analysis of the bacterial community in the lumen contents of different treated transplantation rats. Bacterial community profiles were generated from the DNA of luminal samples using the universal PCR primers 8f and 798r. N, normal control group; I, Syn group; P, PBS group; C, CO group; F, FO group. t, transplanted intestine; i, ileum; c, colon.

**Figure 2 pone-0020460-g002:**
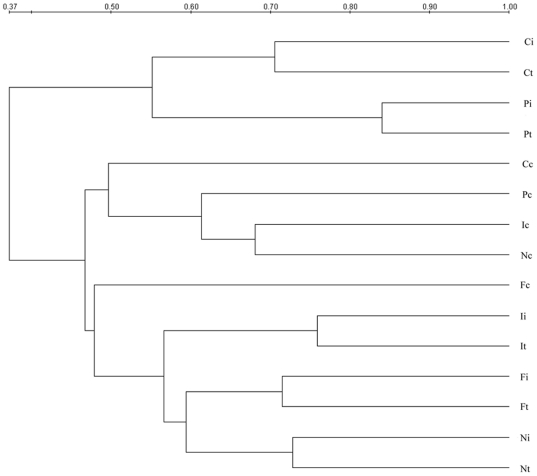
Dendrogram derived from DGGE analysis based on UPGMA clustering algorithm. The scale shown here represents percentage similarity between DGGE profiles.

**Figure 3 pone-0020460-g003:**
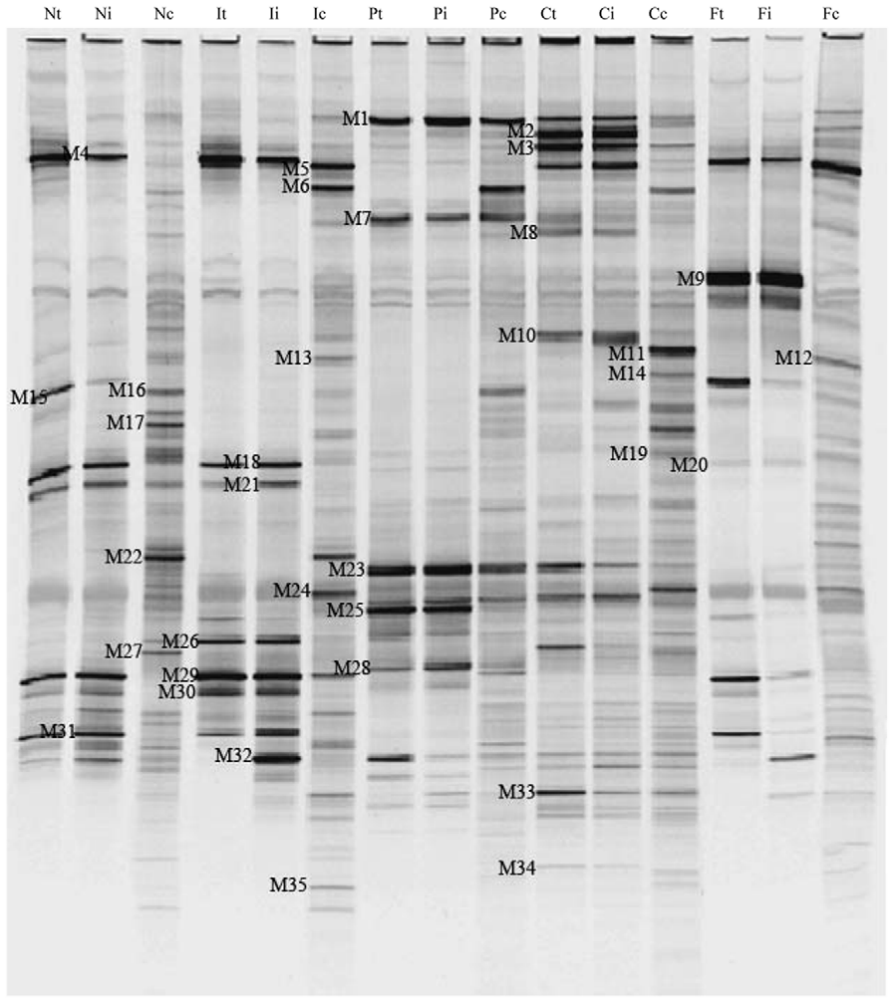
DGGE profiles of the V3 region of the 16S rDNA in the microbiota of mucosal samples. Letters in the image were the same as those in Figure 1.

To characterize gut microbiota shifts in CR rats and determine the effects of fish oil on the restoration of gut microbiota after allogeneic transplantation, we performed a global survey of the microbiota in intestinal contents and mucosal samples from the grafted intestine, recipients' native ileum and colon at day 190 posttransplant. Genetic fingerprints of the intestinal bacterial communities generated by PCR-DGGE analysis showed shifts of the bacterial composition and diversity in luminal contents (Figure 1) and intestinal mucosa (Figure 3) during chronic rejection after intestinal transplantation. As shown in Figure 2, the similarity indices of luminal microbiota in graft and host's ileum of each group ranged from 71% to 84%, indicating higher similarity in gut microbiota in transplanted intestines and native ileum of CR rats. Clustering analysis based on the similarity indices showed that Syn group clustered together with normal group and FO group, and PBS group and CO group clustered in another branch. It indicated that the microbial composition in the graft and host's ileum lumen had a significant variation during chronic rejection after intestinal transplantation. The luminal microbiota in FO-fed animals was closer to those in Syn and normal control rats (54% and 59%, respectively), which revealed the effects of fish oil on the recovery of intestinal luminal microbiota after allogeneic transplantation. The DGGE fingerprint showed that the band number from the colon contents was much larger than those of the graft and ileal luminal samples, indicating an increasing bacterial diversity. The microbial composition in the colonic contents was different from that in the graft and ileal luminal samples, which clustered in a single branch. The variation of the luminal microbiota in the colon was almost consistent with those in the grafted intestine and host's ileum in different groups. In FO group, the DGGE profile of the luminal microbiota in the colon was also similar to that of Syn group and normal group.

**Figure 4 pone-0020460-g004:**
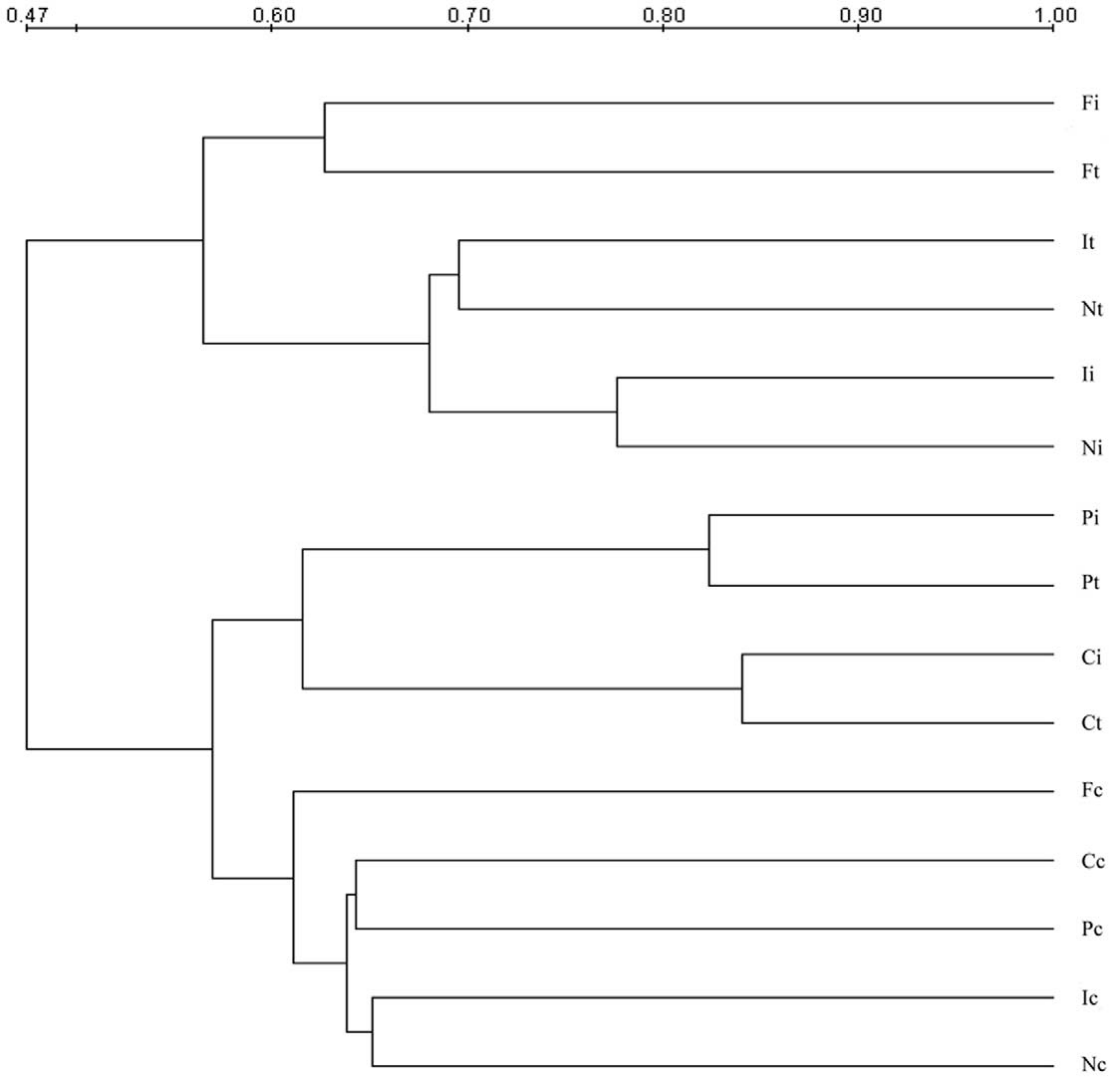
Cluster analysis of DGGE profiles of the mucosal samples. The dendrogram was constructed using UPGMA method.

The similar shifts of the microbial composition were found in the DGGE profiles of mucosa-related microbiota (Figure 3 and 4). As shown in Figure 4, the similarity indices of mucosa-associated microbiota in graft and host's ileum of each group was also at a high level, ranging from 71% to 84%. These data demonstrated that the similar bacteria resided in graft and ileal mucosa in CR rats, which was consistent with that in the lumen. Clustering analysis showed that the similarity indices of mucosa-associated microbiota in graft and native ileum was 57% in FO and normal control group, which was much higher than that between PBS group and normal rats (47%). The shifts of colon mucosa-associated microbiota in FO group also resembled those of the colon contents in Syn group and normal control group.

These results demonstrated that the gut microbiota had significant changes in CR rats after small bowel transplantation. Further analysis in dendrogram was noted that the gut microbiota in FO group was similar to that in normal control and Syn groups but not PBS group and CO-fed animals. It suggested that long-term fish oil treatment resulted in significantly different composition of the intestinal microbiota and enhanced the recovery of gut microbiota from a chaotic status during CR after intestinal transplantation.

### Impact of fish oil administration on bacterial colonization during chronic rejection after intestinal transplantation

To further investigate bacterial colonization in the gut during chronic rejection after intestinal transplantation, bacterial species were identified by comparative sequence analysis of 16S rRNA gene fragments from the DGGE profiles (Table 1 and 2). The DGGE profile from the luminal samples (Figure 1 and Table 1) showed that the most prominent bands corresponded closely to *Weissella paramesenteroides* (band F4), *Lactobacillus sp.* (band F5, F19, F28 and F30) in the graft and native ileal contents in Syn group. Instead of that, the major bands corresponding to *Bacteroides fragilis* (band F1), *Parabacteroides distasonis* (band F10), *Escherichia coli* (band F24) *Acidovorax ebreus* (band F26) and *Clostridium botulinum* (band F27) were found and had high abundance in the graft and host's ileal lumen in PBS and CO groups. As compared with Syn group, the intensity of bands corresponding to *Weissella paramesenteroides* (band F4), and *Lactobacillus sp* (band F5 and F30) in the PBS and CO group were decreased, while *Bacteroides thetaiotaomicron* (band F2) and *Bacteroides uniformis* (band F3) were increased after corn oil treatment. It demonstrated that the species of dominant bacteria in the intestinal lumen markedly varied in CR animals after intestinal transplant, while the relative abundance of *Lactobacillales* species decreased, and those of *Escherichia coli* and *Bacteroides* species increased during CR. In FO-fed rats, the major bacteria including *Weissella paramesenteroides* (band F4) and *Lactobacillus johnsonii* (band F5, F19 and F30) in the intestinal lumen were identical to those in normal control and syngenic transplanted animals. The results revealed that the long-term fish oil supplementation was beneficial for the resident of normal dominant bacteria in the graft and ileal lumen in CR after intestinal transplantation.

**Table 1:** Similarities of 16S rDNA gene sequences of luminal microbiota with closest match sequences retrieved.

Band	Closest relative	% similarity	clones	Accession no.
L1	Bacteroides fragilis	100	YCH46	NC_006347.1
L2	Bacteroides thetaiotaomicron	98	VPI-5482	NC_004663.1
L3	Bacteroides uniformis	95	ATCC 8492	NZ_DS362249.1
L4	Weissella paramesenteroides	95	ATCC 33313	NZ_GG697131.1
L5	Lactobacillus johnsonii	100	ATCC 33200	NZ_GG670123.1
L6	Prevotella copri	98	DSM 18205	NZ_GG703856.1
L7	Clostridium cellulolyticum	90	H10	NC_011898.1
L8	Bacteroides stercoris	92	ATCC 43183	NZ_DS499676.1
L9	Helicobacter winghamensis	93	ATCC BAA-430	NZ_GG661973.1
L10	Parabacteroides distasonis	100	ATCC 8503	NC_009615.1
L11	Bacteroides sp.	96	D20	NZ_GG730109.1
L12	Lactobacillus jensenii	100	SJ-7A-US	NZ_GG704690.1
L13	Oribacterium sp. oral taxon	94	078 str.F0262	NZ_GG729933.1
L14	Bacteroides cellulosilyticus	95	DSM 14838	NZ_EQ973490.1
L15	Lactobacillus helveticus	95	DSM 20075	NZ_GG700755.1
L16	Clostridium sp.	96	M62/1	NZ_GG730316.1
L17	Prevotella oris	96	F0302	NZ_GG703886.1
L18	Clostridium saccharolyticum	96	WM1	NC_014376.1
L19	Lactobacillus coleohominis	92	101-4-CHN	NZ_GG698808.1
L20	Clostridium bolteae	100	ATCC BAA-613	NZ_DS480694.1
L21	Lactobacillus salivarius	93	ATCC 11741	NZ_GG693227.1
L22	Shigella flexneri	95	5 str. 8401	NC_008258.1
L23	Prevotella sp. oral taxon	97	472 str. F0295	NZ_GG704830.1
L24	Escherichia coli	97	MS 146-1	NZ_GG772052.1
L25	Porphyromonas gingivalis	95	ATCC 33277	NC_010729.1
L26	Acidovorax ebreus	95	TPSY	NC_011992.1
L27	Clostridium botulinum	95	E3 str. Alaska E43	NC_010723.1
L28	Lactobacillus reuteri	98	CF48-3A	NZ_GG693685.1
L29	Clostridium perfringens	98	ATCC 13124	NC_008261.1
L30	Lactobacillus antri	99	DSM 16041	NZ_GG700732.1
L31	Clostridium leptum	95	DSM 753	NZ_DS480348.1
L32	Syntrophus aciditrophicus	99	SB	NC_007759.1
L33	Clostridium bartlettii	100	DSM 16795	NZ_DS499553.1
L34	Syntrophobacter fumaroxidans	93	MPOB	NC_008554.1
L35	Clostridium beijerinckii	98	NCIMB 8052	NC_009617.1
L36	Desulfovibrio desulfuricans	96	ATCC 27774	NC_011883.1
L37	Bacteroides coprophilus	93	DSM 18228	NZ_EQ973630.1

**Table 2:** Similarities of 16S rDNA gene sequences of mucosal-related microbiota with closest match sequences retrieved.

Band	Closest relative	% similarity	clones	Accession no.
M1	Bacteroides fragilis	99	YCH46	NC_006347.1
M2	Bacteroides thetaiotaomicron	99	VPI-5482	NC_004663.1
M3	Bacteroides uniformis	95	ATCC 8492	NZ_DS362249.1
M4	Lactobacillus johnsonii	100	ATCC 33200	NZ_GG670123.1
M5	Prevotella copri	98	DSM 18205	NZ_GG703856.1
M6	Clostridium cellulolyticum	90	H10	NC_011898.1
M7	Parabacteroides distasonis	99	ATCC 8503	NC_009615.1
M8	Bacteroides sp.	98	D20	NZ_GG730109.1
M9	Lactobacillus jensenii	99	SJ-7A-US	NZ_GG704690.1
M10	Fusobacterium nucleatum	95	ATCC 10953	NZ_CM000440.1
M11	Coprococcus comes	92	ATCC 27758	NZ_GG662006.1
M12	Ruminococcus lactaris	99	ATCC 29176	NZ_DS990170.1
M13	Bacteroides cellulosilyticus	97	DSM 14838	NZ_EQ973490.1
M14	Lactobacillus helveticus	95	DSM 20075	NZ_GG700755.1
M15	Lactobacillus ultunensis	97	DSM 16047	NZ_GG693258.1
M16	Prevotella oris	98	F0302	NZ_GG703886.1
M17	Eubacterium rectale	91	ATCC 33656	NC_012781.1
M18	Lactobacillus coleohominis	92	101-4-CHN	NZ_GG698808.1
M19	Clostridium bolteae	100	ATCC BAA-613	NZ_DS480694.1
M20	Lactobacillus acidophilus	95	ATCC 4796	NZ_GG669569.1
M21	Lactobacillus salivarius	93	ATCC 11741	NZ_GG693227.1
M22	Prevotella sp. oral taxon	97	472 str. F0295	NZ_GG704830.1
M23	Escherichia coli	97	MS 146-1	NZ_GG772052.1
M24	Porphyromonas gingivalis	93	ATCC 33277	NC_010729.1
M25	Clostridium botulinum	97	E43	NC_010723.1
M26	Lactobacillus reuteri	98	CF48-3A	NZ_GG693685.1
M27	Prevotella bergensis	98	DSM 17361	NZ_GG704785.1
M28	Clostridium perfringens	100	ATCC 13124	NC_008261.1
M29	Lactobacillus antri	96	DSM 16041	NZ_GG700732.1
M30	Pediococcus acidilactici	94	DSM 20284	NZ_GL397069.1
M31	Lactobacillus vaginalis	98	ATCC 49540	NZ_GG693419.1
M32	Clostridium bartlettii	100	DSM 16795	NZ_DS499553.1
M33	Clostridium beijerinckii	98	NCIMB 8052	NC_009617.1
M34	Desulfovibrio desulfuricans	94	ATCC 27774	NC_011883.1
M35	Bacteroides coprophilus	92	DSM 18228	NZ_EQ973630.1

The dominant bands from mucosal samples were similar to those in the luminal samples by sequence analysis (Figure 3 and Table 2). In the graft and ileal mucosa of Syn group, the prominent bands were closely related to *Lactobacillus sp.* (band M4, M18, M21, M26, M29 and M31) at 190 days after intestinal transplant, which was almost consistent with those in normal rats. The intense bands corresponding to *Bacteroides fragilis* (band M1), *Escherichia coli* (band M23), *Clostridium sp*. (band M25 and M28) and *Parabacteroides distasonis* (band M7) were found in the profile of PBS-treated rats. The dominant bacteria presented in CO-fed animals were similar to those in PBS group. These results indicated that the predominant bacteria in the graft and ileal mucosa also had significant changes in CR rats after intestinal transplantation. Three bands corresponding to *Lactobacillus johnsonii* (band M4), *Lactobacillus antri* (band M29) and *Lactobacillus vaginalis* (band M31) appeared and had high abundance in the graft's mucosa of FO group, which was consistent with those of control group and Syn group. Meanwhile, *Lactobacillus jensenii* (band M9) was present and became one of the most abundant bacteria in the grafted and ileal mucosa of FO group. Although the dominant bacteria species in FO group were not completely identical to those in control and Syn groups, these bacteria belonged to a same genus *Lactobacillus*. It revealed that fish oil could retrieve the alteration of mucosa-associated microbiota during chronic rejection after small bowel transplantation.

### Alteration of the predominant microbes in the gut of CR animals by fish oil treatment

**Figure 5 pone-0020460-g005:**
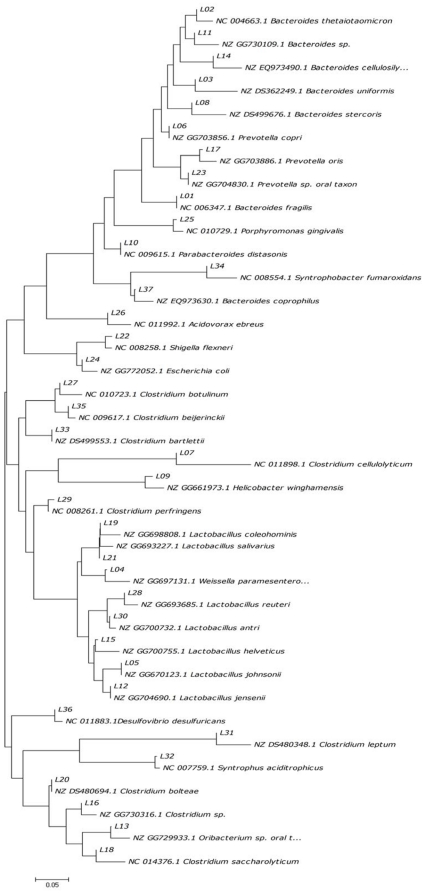
Neighbor-joining tree showing phylogenetic relationships of 16S rDNA sequences cloned from intestinal contents to closely related sequences from GenBank. The tree was constructed using the neighbor-joining method. The bar indicates 5% sequence divergence.

**Figure 6 pone-0020460-g006:**
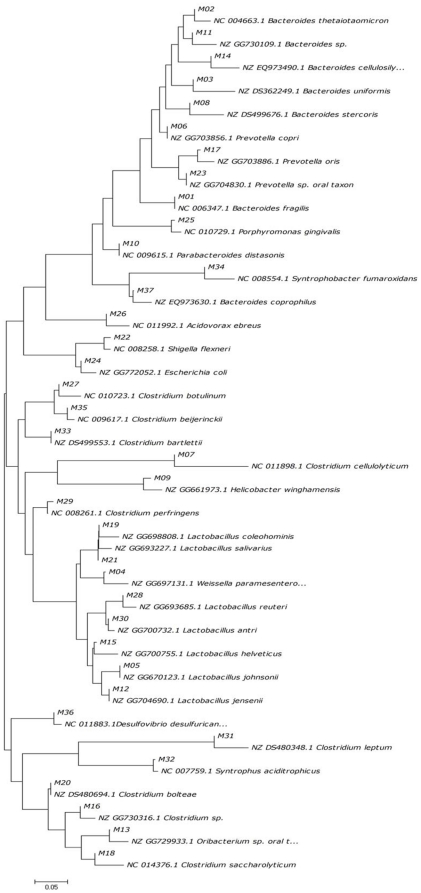
Phylogenetic tree for partial sequences of cloned 16S rDNA fragments and the most closely related bacteria. The tree was constructed by neighbor-joining analysis.

All the partial 16S rDNA sequences representing the respective excised DGGE bands were aligned and phylogenetically analyzed (Figure 5 and 6). The neighbor-joining analysis showed that the major sequences in luminal contents were divided into three clusters (Figure 5), which were *Bacteroidetes* (12 sequences), *Firmicutes* (total 18 sequences in which 10 of *Clostridia* and 8 of *Lactobacillales*), *Proteobacteria* (7 sequences), which in mucosal samples were also divided into three clusters (Figure 6). The dominant bacteria in Syn and FO group belonged to *Lactobacillales*, which in PBS and CO group clustered to *Bacteroides*, *Clostridia* and *Enterobacteriaceae*. The results suggested that the *Bacteroides*, *Clostridia* and *Enterobacteriaceae* species greatly increased and became predominant instead of *Lactobacillales* bacteria in CR rats. These data confirmed that long-term dietary fish oil would enhance the colonization of *Lactobacillales* bacteria in intestines and contribute to the recovery of gut microbiota during chronic rejection after intestinal transplantation.

### Fish oil treatment improved intestinal epithelial TJ ultrastructure

**Figure 7:**
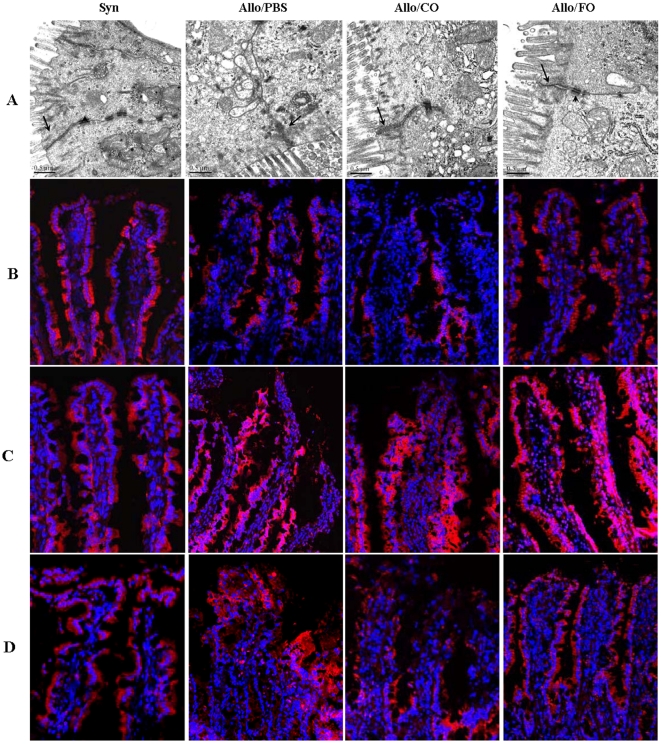
Fish oil prevented the alteration of TJ ultrastructure and redistribution of TJ proteins in intestinal transplant rats. (A): Transmission electron microscopic analysis was performed to investigate changes in the ultrastructure of TJ during chronic rejection. Arrows indicated apical TJ and arrows heads depicted desmosomes. In PBS and CO groups, the electron dense materials decreased significantly, indicating disruption of TJ ultrastructure both in the allograft. In addition, desmosomes disappeared in the two groups. In contrast, fish oil preserved the ultrastructure of TJs. In FO group, TJs and desmosomes displayed intact structure as those in syngeneic group. Bars in the images were 0.5 µm. (B): Examination of the tight junction protein occludin by immunofluorescency. The allograft intestine was subjected to immunostaining with the corresponding antibody. And the distribution of TJ proteins in the epithelium was examined by immunofluorecency. (C): Immunolocalization of claudin-1 in the allograft intestine in intestinal transplant rats. The allograft intestine was subjected to immunostaining with the claudin-1 antibody, and images were captured with a confocal scanning microscope. (D): Immunofluorescence staining for claudin-3. Fish oil prevented redistribution of claudin-3 in the allograft intestine.

The intact barrier of epithelia is essential for physiological homeostasis and defense against extrinsic antigens [29]. Tight junctions are essential for the barrier function of the epithelia. To investigate whether intestinal transplant had effect on tight junction, we first assessed changes in the ultrastructure of TJs with Transmission electron microscopy (TEM). TEM examination revealed marked structural abnormalities in TJ region of the epithelium in allogeneic rats. Altered TJ ultrastructure was more pronounced in tissues from PBS group and coin oil treated rats, in which less electron density materials were present at TJs and some microvilli were lost, indicating that TJ ultrastructure was destroyed (Figure 7A and Figure S1). However, much electron density materials were observed in the loci of TJs, demonstrating intact TJ ultrastructure in syngeneic group. And the desmosomes also displayed intact structure. Additionally, large number of microvilli was well oriented in this group. Most strikingly, Administration of fish oil alleviated the distortion of TJ ultrastructure. The TJs and desmosomes demonstrated normal structures as those in the syngeneic group.

### Fish oil prevented redistribution of TJ proteins

In order to characterize the barrier damage during shifts in gut microbiota induced by chronic rejection, we performed immunofluorescency to examine the expression and subcellular localization of TJ structural proteins. The evidences have suggested that occludin is involved in the barrier and fence functions of TJs [30], [31]. In addition, establishment of TJ strands depends on claudins, especially claudins 1 and 3 which can form continuous TJ strands [30]. Thus, we investigated changes in the localization of occludin, claudin-1 and 3 in intestinal transplant rats by immunostaining. The immunofluorescence assay demonstrated an intact network of occludin which was mainly localized in the tight junction region of the surface epithelial cells in the allograft intestine (Figure 7B) and recipients' native ileum (Figure S2) in syngeneic group. And the localization of occludin showed no obvious gradient in staining along the crypt-to-villus axis. In contrast, occludin staining appeared to be decreased and discontinuous in the tissues of the PBS group and CO-fed animals (Figure 7B and Figure S2). The signal intensity of occludin was much weaker at the apicalmost tips of the villi than that in the crypt epithelium both in the allografts and recipients' native ileum. Furthermore, we found that fish oil treatment could modulate the expression of occludin, leading to a significant increase in the expression of occludin in tight junction region as compared to PBS and CO rats (Figure 7B and Figure S2), which indicated that fish oil could effectively prevent the redistribution of tight junction proteins in intestinal transplant. Claudin-1 was distributed at the villous tip and along the lateral membranes of epithelial cells in the allograft intestine (Figure 7C) and recipients' native ileum (Figure S3) in syngeneic group. The pattern of claudin-1 distribution was altered during chronic rejection. The immunofluorescent staining of claudin-1 at the villous tips was greatly decreased both in the PBS and CO groups. However, fish oil effectively inhibited the relocalization of claudin-1 and the expression pattern was similar as that in the syngeneic group (Figure 7C and Figure S3). Immunofluorescent staining revealed that claudin-3 was not restricted to the apical pole of epithelial cells, and it was also detected along the crypt-villus axis of the intestinal epithelium (Figure 7D and Figure S4). Chronic rejection also led to a decline in the expression of claudin-3 at the apical epithelial cells in PBS and CO groups. And this redistribution was alleviated by fish oil. In addition, using biotin tracer experiment we found that TJ barrier function was disrupted in the allograft intestine in PBS group and CO-fed animals (Figure S5). Whereas, fish oil prevented TJ barrier function disruption.

### Fish oil attenuated mucosal damage in intestinal transplant rats

The intestinal mucosa was examined by H& E staining. Significant changes of the intestinal grafts were noticed 190 days post transplant. The intestinal grafts from the PBS group showed severe mucosal damage characterized by marked villous flattening and focal mucosal necrosis in the allografted intestine (Figure S6A). And significant infiltration with inflammatory cells and massive hemorrhage were also found in the lamina propria. Similarly, the damage of mucosa was present in the allografted intestine in CO rats. In contrast, it showed normal mucosal architecture in the syngeneic group. Interestingly, we found that rats fed with fish oil showed significant attenuation of mucosal injury (Figure S6A). The intestine from FO-fed animals was well protected with normal villous architecture and little inflammatory cell infiltration. Furthermore, the colon of the recipients was assessed. As shown in Figure S6B, chronic rejection resulted in lifting and sloughing of colonic surface epithelium, especially in CO group, extensive superficial ulceration with exposed lamina propria was found. However, the epithelium was restored after fish oil treatment, in which group the epithelium had taken on a normal columnar appearance (Figure S6B).

## Discussion

In this study, we observed significant alterations in the composition of intestinal microbiota and disruption of epithelial integrity in CR. Furthermore, exogenous administration of fish oil promotes restoration of the normal gut microbiota and intestinal tight junction in CR rats. The intestinal transplanted rats displayed major historical characteristics of chronic rejection. Tissue damage was most pronounced in the allografts and recipients' native ileum. Our data suggested the histological characteristics of intestinal epithelial injury and chronic inflammation in chronic rejection. Besides, here we found that administration of fish oil to intestinal transplant rats was associated with an improvement of the mucosal structure and the intestinal epithelial integrity was well protected. Moreover, inflammatory cells infiltration was markedly reduced by fish oil. It is well known that enteric pathogenic bacteria compete with the endogenous microbiota and that infections are more common when the normal intestinal microbiota is lost. We examined the status of the gut microbiota in the graft, recipients' native ileum and colon in CR rats and found dramatic differences in CR rats (Figures 1–7) compared with syngeneic transplanted animals and normal rats. The rats of chronic rejection were accompanied by the shifts of gut microbiota towards *Escherichia coli*, *Bacteroides* and *Clostridium* groups. Meanwhile, the relative abundance of *Lactobacillales* bacteria in the intestines had a significant decrease in CR rats. Similar changes were showed in experimental models of intestinal inflammation [32], [33] and in patients with IBD [34], [35], which indicated that these alterations are caused by inflammation.

Phylogenetic analysis ultimately determined the composition of bacteria in CR rats. Comparison of phylogenetic data showed that CR rats had more *Escherichia coli*, *Bacteroides spp*. and *Clostridium* groups than syegeneic transplanted rats and normal rats (Figure 5 and 6). Patients with active intestinal inflammation displayed accumulation of commensal *Escherichia coli* or *Bacteroides spp.* at inflamed tissue sites [36], [37]. These bacterial groups also suspected to trigger intestinal inflammation in acute graft-versus-host-disease after bone marrow transplantation [38]. The extensive overgrowth of *Escherichia coli* indicates that fast-growing bacteria may take advantage of increased sugar and oxygen concentrations induced by inflammation and tissue damage [32]. This is in consistent with the proinflammatory potential of *Escherichia coli*, and *Bacteroides spp.* in various models of intestinal inflammation [32]. Notably, increased intestinal *Escherichia coli and Bacteroides spp.*, particularly in the intestinal mucosa, were significantly associated with intestinal pathology and disruption of tight junction in CR rats. Our previous results showed that enteropathogenic *Escherichia coli* could disrupt the tight junction in intestinal epithelial cells *in vitro* [39] and change distribution of occludin and ZO-1 in tight junction membrane microdomains *in vivo* [40]. These results indicated that the disruption of the intestinal epithelial TJ in chronic rejection might result from overgrowth of *Escherichia coli* in intestinal microbiota.

The number and position of bands in DGGE profiles showed that the molecular pattern of the gut microbiota of CR rats treated by fish oil were similar to the reference pattern of syngeneic transplanted and normal animals, showing 54% and 59% concordance in the luminal contents, and 57% and 57% in the intestinal mucosa from graft and host's ileum, respectively. We observed similar alterations in bacterial composition of the small intestinal lumen and mucosa in CR rats treated with fish oil. Fish oil treated rats showed a significant decrease of gut bacterial proportions of *Escherichia coli*, *Bacteroides spp.* and *Clostridium* groups as compared to CR rats. Meanwhile, the colonization of *Lactobacillales* bacteria in FO-fed rat intestines was enhanced significantly. It is well known that enteric pathogenic bacteria compete with the endogenous microbiota and that enteric infections are more common in settings where the normal intestinal microbiota is lost or disrupted. In this study, long-term fish oil treatment had greatly increased the abundance of *Lactobacillales* bacteria in small intestines of CR rats, which indicated that fish oil might enhance the growth and adhesion of *Lactobacillus* to intestinal surfaces and also appear to inhibit the colonisation and infection of the gut opportunistic pathogen such as *Escherichia coli*. The recovery of the normal gut microbiota by fish oil was accompanied by the improvement of intestinal barrier and epithelial TJ ultrastructure.

Noticeably, ultrastructural observations revealed epithelial cell abnormalities, showing that the tight junctional structure was disrupted in CR rats. Along the same line, we found that chronic rejection induced rearrangements of tight junction proteins in TJs. Occludin and claudins immunolocalization in characteristic TJ ‘ring structures’ was severely disrupted, which resulted in the lack of continuity in immunostaining. Marked reduction of TJ proteins may constitute a mechanism for barrier dysfunction in CR rats. Another important finding of the present study was that fish oil had beneficial effect on tight junction in CR rats. The most striking feature of fish oil administration was the recovery of impairment in TJ ultrastructure and prevention of TJ proteins relocalization. Taken together, our study have presented novel evidence that the positive effects of fish oil on intestinal barrier are probably accounted for by hindering movement of occludin and claudins away from the TJs to reestablish barrier function after intestinal transplantation.

The interactions between microbiological communities and the host are complex and poorly understood in CR. And the mechanisms responsible for these beneficial effects of fish oil in CR have not yet been determined. We have previously demonstrated that proinflammatory cytokines affected the epithelial barrier and disrupted the structure of TJs, as well as displaced occludin from membrane microdomains of TJs [13]. Our previous results indicated that n-3 PUFAs play an important role in proinflammatory cytokines-induced permeability defects and epithelial barrier dysfunction by preventing redistribution of TJ proteins [22]. We considered that fish oil mediated regulation of the microbiota was related to changes in the chronic inflammatory state of the intestine in CR. Histological analysis of CR rats revealed inflammatory changes and fish oil inhibited the inflammation in the allografted intestine (Figure S6A). Therefore, the effects of fish oil on the gut microbiota in CR are probably due to an improved gut inflammatory state. However, *in vivo* studies on the effects of PUFAs on the gut microbiota are lacking. Our results demonstrated that the administration of PUFAs had positive influence in imbalance of the gut microbiota and enhanced the recovery of commensal gut microbiota in CR, which may be due to that the intake of these fatty acids might influence bacterial adhesion.

In summary, our data showed that CR rats exhibit an altered gut barrier, characterized by the redistribution of tight junction proteins. We found that changes in the gut microbiota of CR rats are dominant by overgrowth of *Escherichia coli*, *Bacteroides spp.* and *Clostridium spp.* and decreased *Lactobacillales*. In addition, fish oil improved the tight junctions through expression of tight junction proteins. Supplementation of fish oil in CR rats favored the growth of commensal bacteria and enhanced restoration of gut microbiota lost due to CR of intestinal transplantation. The restoration of intestinal epithelial TJ was also closely associated with the reestablishment of intestinal normal microbiota in CR rats treated by fish oil. Based on these data, we suggest that orally administered fish oil may have therapeutic potential against CR in intestinal transplantation.

## Supporting Information

Figure S1 **Changes of TJ ultrastructure in the recipients' native ileum in intestinal transplant rats.** TJ ultrastructure was examined by Transmission electron microscopy.
(TIF)

Figure S2 **Occludin localization in the recipients' native ileum in intestinal transplant rats. **Frozen sections of the recipients' native ileum were stained with the antibody to claudin-1.
(TIF)

Figure S3 **Localization of the TJ protein claudin-1 in the recipients’ native ileum in intestinal transplant rats.** Claudin-1 localization was investigated by immunostaining.
(TIF)

Figure S4 **Localization of claudin-3 in the recipients' native ileum in intestinal transplant rats.** The co-staining of claudin-3 (red) and DAPI (blue) images were presented.
(TIF)

Figure S5 **Fish oil protected injury of intestinal barrier permeability in intestinal transplant rats.** The tracer molecule biotin was held to the luminal border of the intestine in syngeneic group. While biotin fluorescent staining penetrates the epithelium into tissue in PBS and CO-Fed animals. In FO group biotin was held to the luminal border as that in the syngeneic group. Nucleus was stained with DAPI (blue).
(TIF)

Figure S6 **Histological appearance of the allograft intestine and recipients' native ileum (A) and colon (B).** (A) Representative photomicrographs of the allograft intestine and recipients' native ileal mucosa in rats after intestinal transplant (magnification ×100). Widespread destruction of villi was observed in PBS and CO group. And fish oil prevented mucosal destruction. (B) Histopathologic findings of the rat colon (magnification ×400). Sloughing of epithelium from the tips of the colon was present in CO group. And fish oil preserved mucosal architecture.
(TIF)

Text S1 **Suppenmentary Material and Methods, including Reagents, Histopathology, Extraction of total DNA in samples, PCR amplification of bacterial DNA, DGGE analysis, Sequencing of DGGE bands from gels, Transmission electron microscopy, Immunofluorescence staining of tight junction proteins, and TJ barrier function measurement used in this study.**
(DOC)
