# Supplementary figures and images for: Fish Oil Enhances Recovery of Intestinal Microbiota and Epithelial Integrity in Chronic Rejection of Intestinal Transplant
Source: PLoS One. 2011 Jun 17;6(6):e20460. doi: 10.1371/journal.pone.0020460 (PMC3117781; doi:10.1371/journal.pone.0020460)

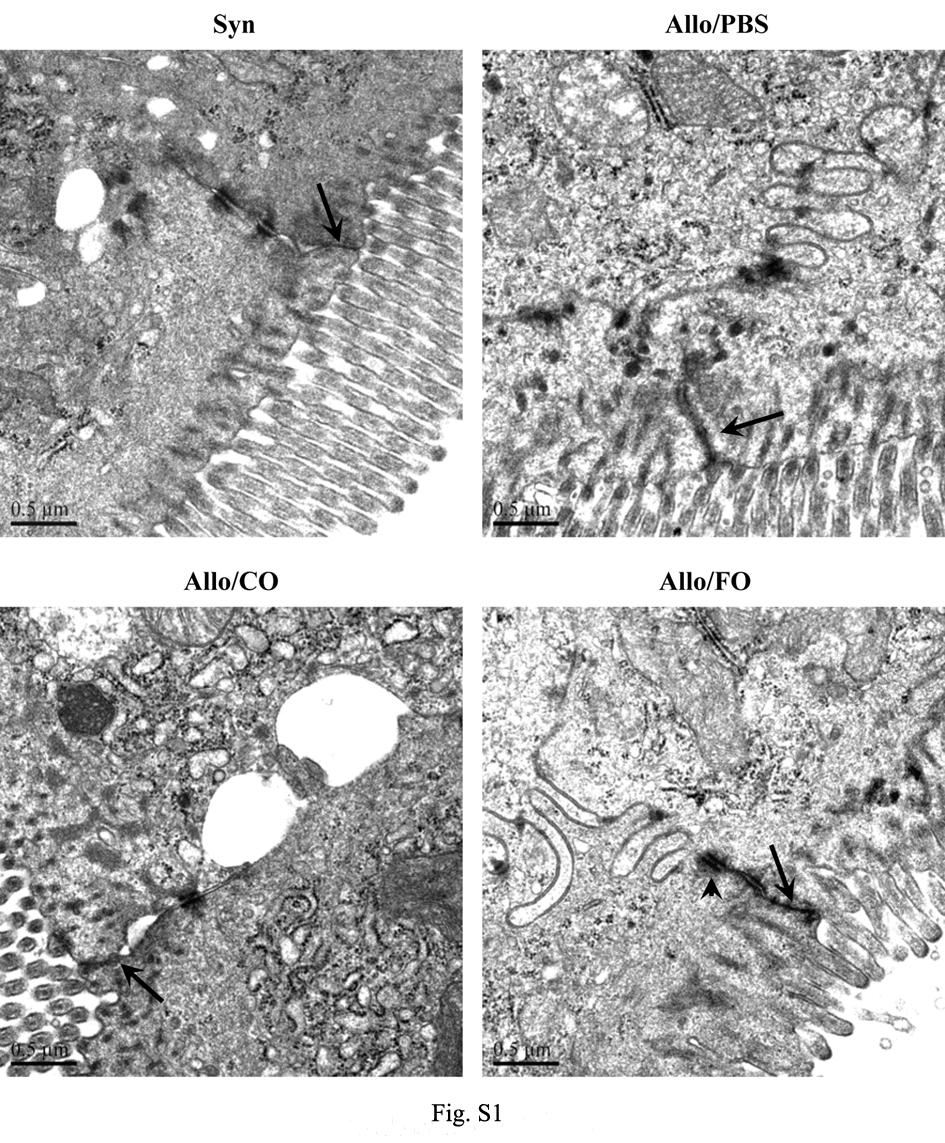

Supplement: Figure S1 — Changes of TJ ultrastructure in the recipients' native ileum in intestinal transplant rats. TJ ultrastructure was examined by Transmission electron microscopy. (TIF) [file pone.0020460.s001.tif]

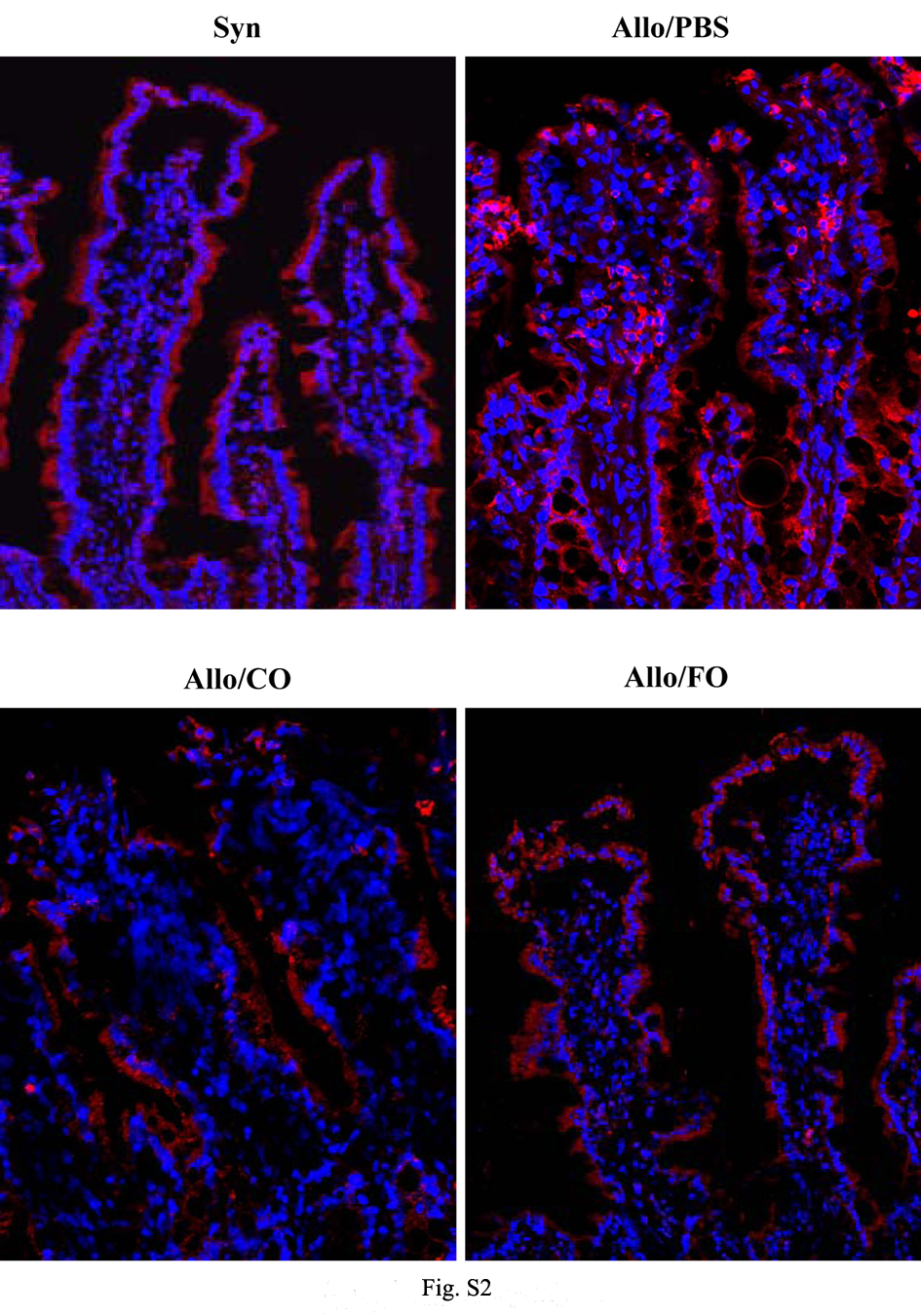

Supplement: Figure S2 — Occludin localization in the recipients' native ileum in intestinal transplant rats. Frozen sections of the recipients' native ileum were stained with the antibody to claudin-1. (TIF) [file pone.0020460.s002.tif]

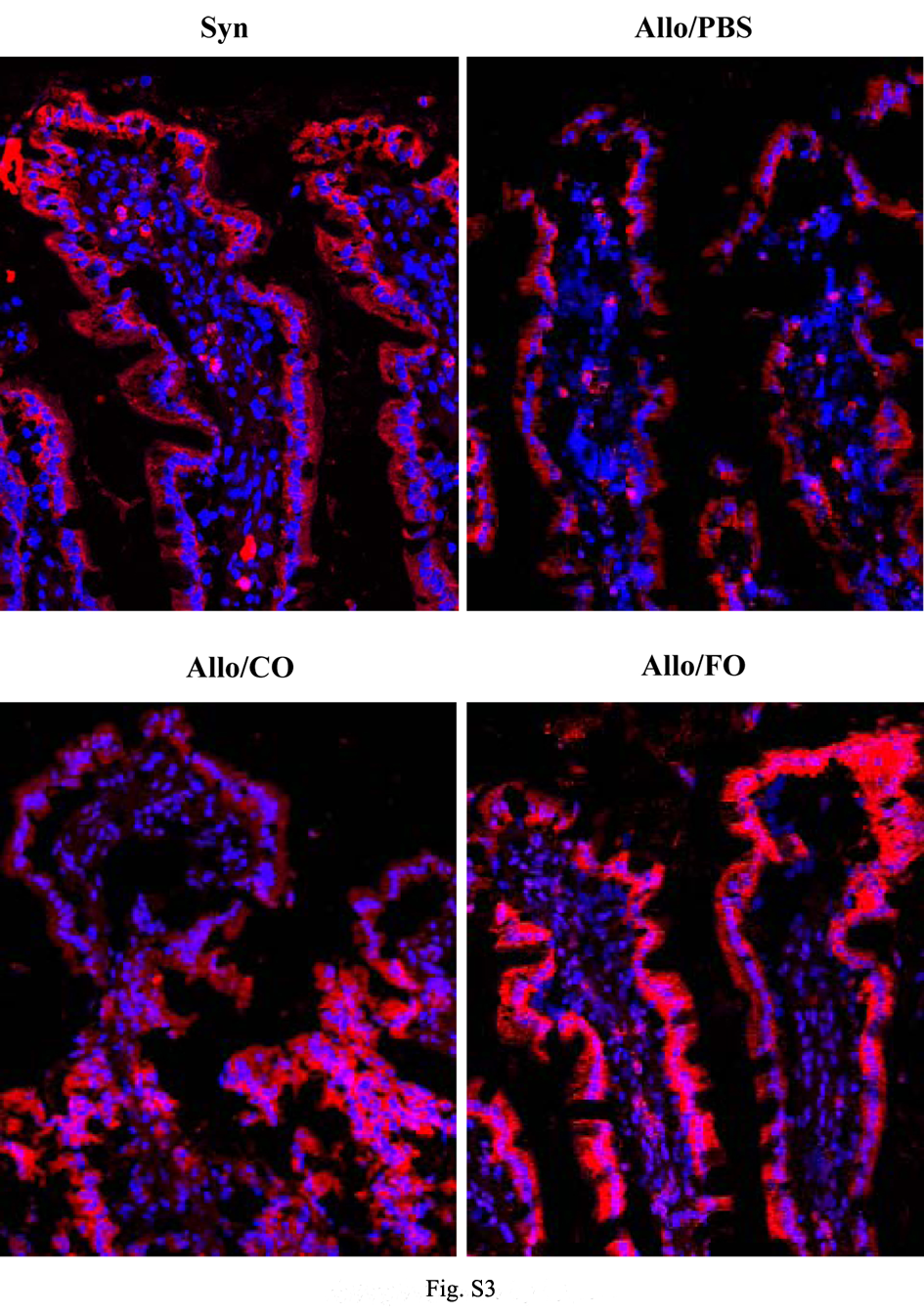

Supplement: Figure S3 — Localization of the TJ protein claudin-1 in the recipients’ native ileum in intestinal transplant rats. Claudin-1 localization was investigated by immunostaining. (TIF) [file pone.0020460.s003.tif]

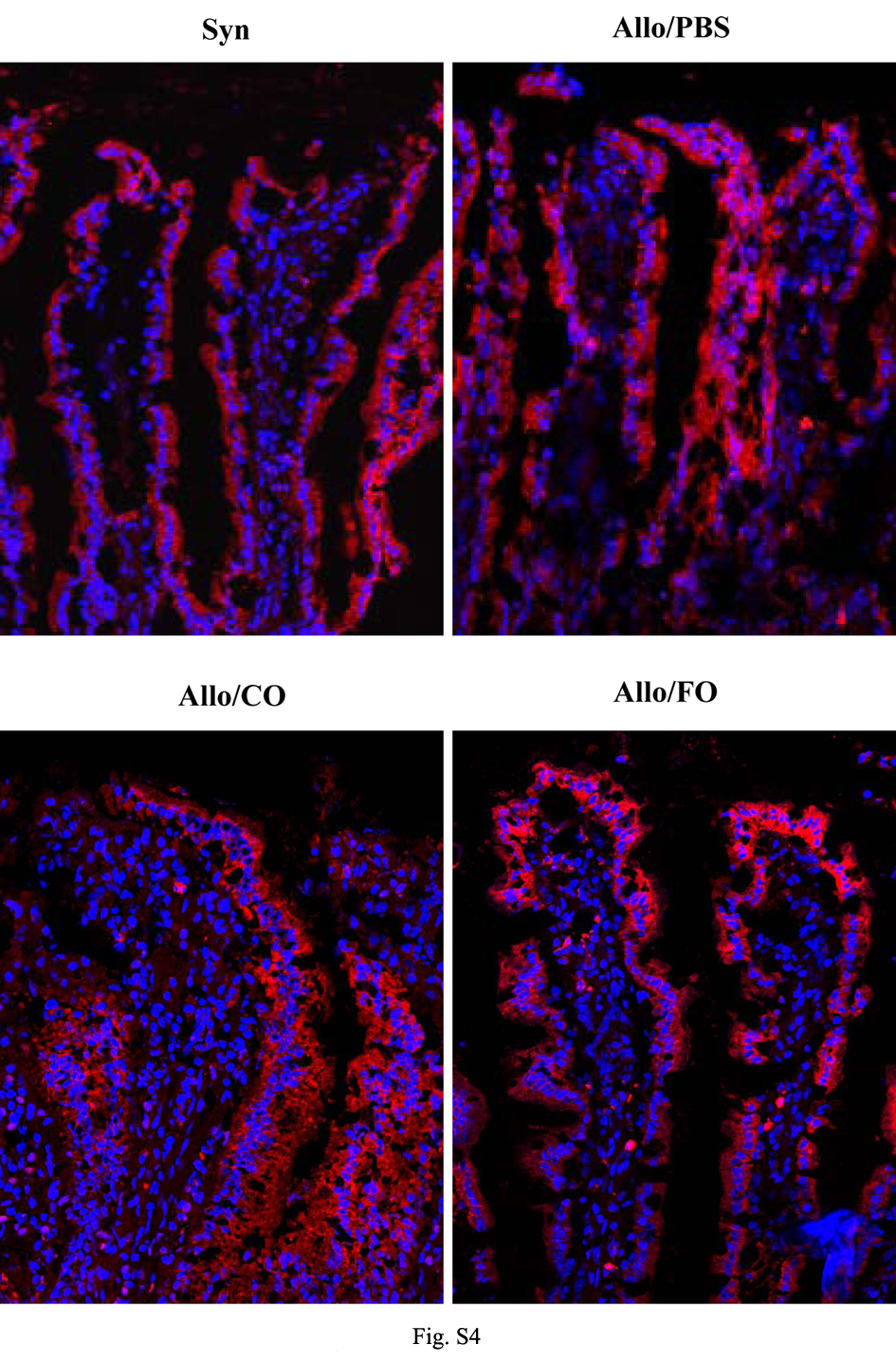

Supplement: Figure S4 — Localization of claudin-3 in the recipients' native ileum in intestinal transplant rats. The co-staining of claudin-3 (red) and DAPI (blue) images were presented. (TIF) [file pone.0020460.s004.tif]

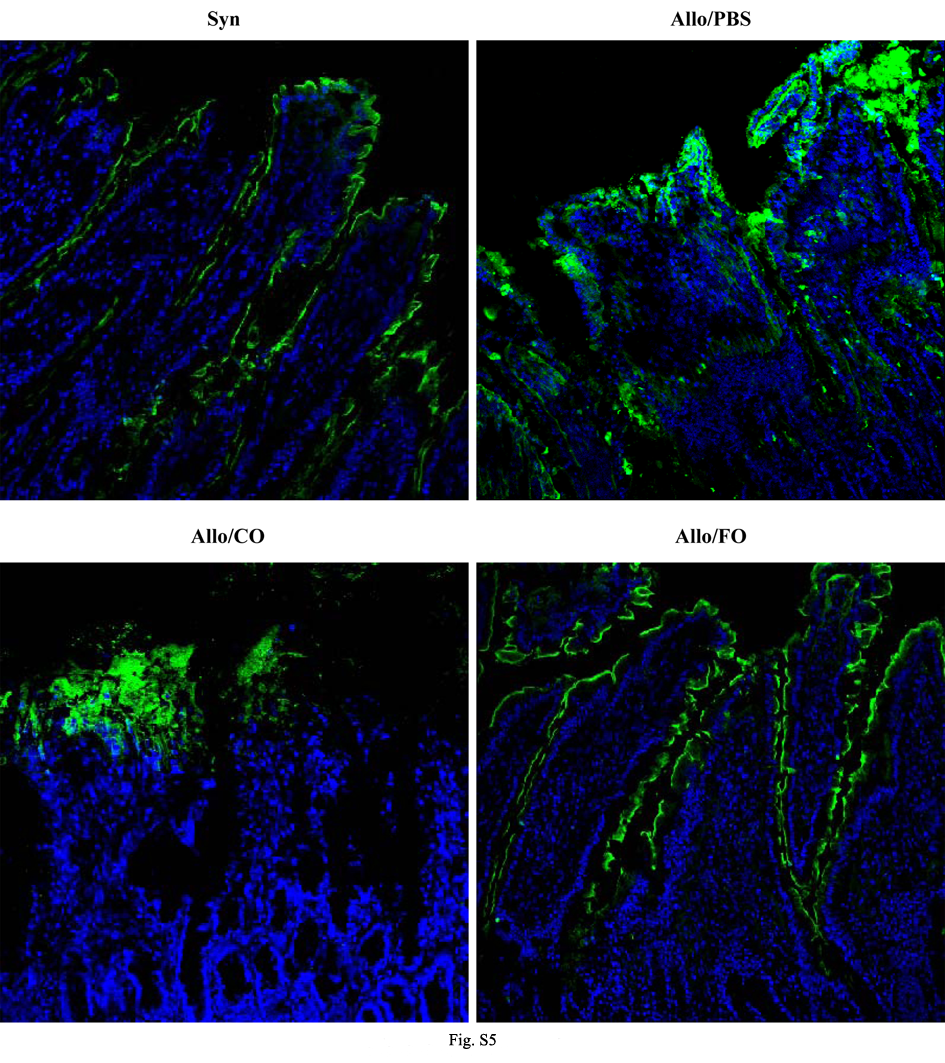

Supplement: Figure S5 — Fish oil protected injury of intestinal barrier permeability in intestinal transplant rats. The tracer molecule biotin was held to the luminal border of the intestine in syngeneic group. While biotin fluorescent staining penetrates the epithelium into tissue in PBS and CO-Fed animals. In FO group biotin was held to the luminal border as that in the syngeneic group. Nucleus was stained with DAPI (blue). (TIF) [file pone.0020460.s005.tif]

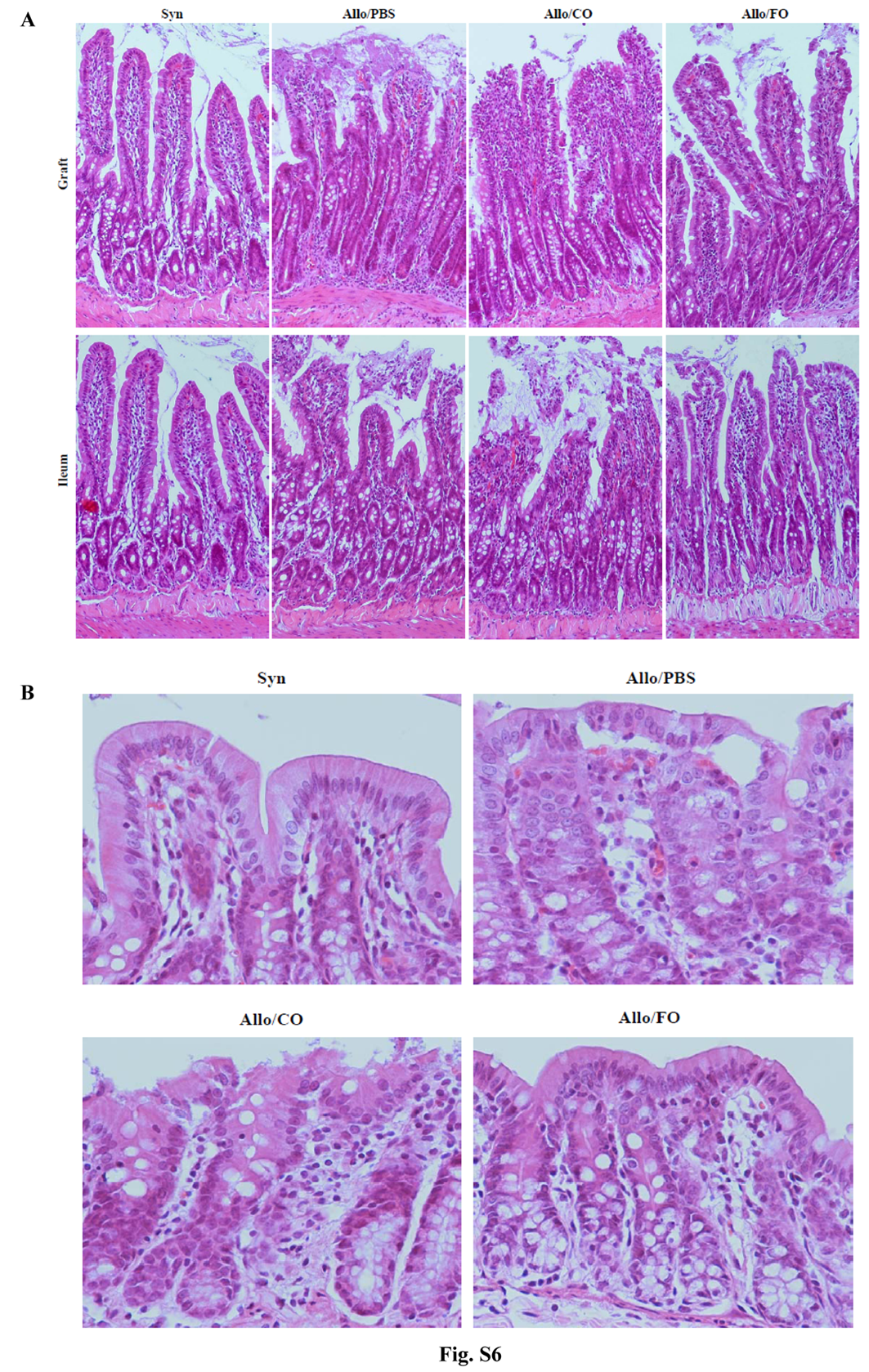

Supplement: Figure S6 — Histological appearance of the allograft intestine and recipients' native ileum (A) and colon (B). (A) Representative photomicrographs of the allograft intestine and recipients' native ileal mucosa in rats after intestinal transplant (magnification ×100). Widespread destruction of villi was observed in PBS and CO group. And fish oil prevented mucosal destruction. (B) Histopathologic findings of the rat colon (magnification ×400). Sloughing of epithelium from the tips of the colon was present in CO group. And fish oil preserved mucosal architecture. (TIF) [file pone.0020460.s006.tif]
