# Supplementary material for: Fish Oil Enhances Recovery of Intestinal Microbiota and Epithelial Integrity in Chronic Rejection of Intestinal Transplant
Source: PLoS One. 2011 Jun 17;6(6):e20460. doi: 10.1371/journal.pone.0020460 (PMC3117781; doi:10.1371/journal.pone.0020460)
Supplement: Text S1 — Suppenmentary Material and Methods, including Reagents, Histopathology, Extraction of total DNA in samples, PCR amplification of bacterial DNA, DGGE analysis, Sequencing of DGGE bands from gels, Transmission electron microscopy, Immunofluorescence staining of tight junction proteins, and TJ barrier function measurement used in this study. (DOC) [file pone.0020460.s007.doc]

**Text S1**

**Reagents**

Rabbit polyclonal antibodies to occludin and claudin-3, and mouse monoclonal antibodies to claudin-1 and 5 were obtained from Zymed Laboratories Inc (San Francisco, CA, USA). The horseradish peroxidase conjugated secondary antibodies were purchased Amersham (Piscataway, NJ, USA). Alexa Fluor-635 goat anti-rabbit and anti-mouse secondary antibodies and DAPI (4’, 6’-diamidino-2-phenylindole) were from Molecular Probes (Eugene, OR, USA). And Super Signal® West Pico Chemiluminescent Substrate was a product of Thermo Scientific (Rockford, IL, USA). Fish oil and corn oil were purchased from Sigma Aldrich (Product Number F8020 and C8267, St. Louis, MO, USA). The major components in fish oil are n-3 polyunsaturated fatty acids (PUFA) including EPA (10-15%) and DHA (8-15%). FK506 was supplied by Astellas Ireland Co. (Astellas, Ireland). All other reagents and compounds used were of analytical grade and obtained from Sigma Aldrich.

**Histopathology**

The grafted intestine and recipients’ native ileum were harvested and placed in 10% neutral buffered formalin. The fixed tissues were paraffin embedded, and 5µm thick sections were cut and stained with hematoxylin and eosin (H & E). The injury and repair of the intestine were assessed by a pathologist in a blinded manner.

**Extraction of total DNA from the luminal contents and tissue samples**

DNA from cryopreserved luminal samples and intetinal tissue were extracted using the QIAamp DNA Stool Mini Kit and QIAamp DNA Mini Kit (QIAGEN Ltd., UK) according to the manufacturer's instructions, respectively. Briefly, samples were suspended in lysis buffer solutions before incubation at 95°C. Proteinase K, buffer ATL, and ethanol were sequentially added. The resultant DNA was adsorbed onto minicolumn membranes, washed, and eluted according to the procedures. The purified DNA were eluted in 200 μl of elution buffer and stored at -20°C until analysis.

**Nested PCR amplification of bacterial DNA**

Nested PCR and touchdown PCR were used for 16S rDNA-V3 amplification. The first PCR applied primers 8f (5’-GGA GAG TTT GAT CA/CT GGC T-3’) and 798r (5’-CCA GGG TAT CTA ATC CTG TT-3’) to amplify a 790-bp fragment of the 16S rDNA gene. The 50 μl PCR mixture contained of 5μl 10×PCR buffer, 1 μl dNTP mixture (2.5 mM each), 1μl each primer (10 pM), 0. 5μl Taq polymerase (5 U/μl), 40.5 μl sterile water, and 1 μl extracted community DNA. The following PCR program was initial denaturation at 94 °C for 5 min, 25 cycles of denaturation at 94 °C for 60 s, annealing at 58 °C for 60 s, and extension at 72 °C for 90 s, and final extension at 72 °C for 5 min followed by cooling to 4°C. Subsequently, using the amplicons of the first PCR as template DNA, the second PCR was performed with primers GC-357f (5’-CGC CCG GGG CGC GCC CCG GGC GGG GCG GGG GCA CGG GGG GCC TAC GGG AGG CAG CAG-3’) and 518r (5’-ATT ACC GCG GCT GCT GG-3’) to amplify V3 region of bacterial 16S rDNA. The reaction mixture was identical to the first PCR amplification except for the primer sets and DNA templates. PCR was performed by using the following protocol: initial denaturation at 94 °C for 5 min, 30 s at 94°C (denaturation), 30 s at 65°C (annealing), and 90 s at 72°C (elongation) with a 1°C touchdown every second cycle during annealing for 20 cycles, followed by 10 cycles with an annealing temperature of 55°C and a final cycle consisting of 5 min at 72°C. PCR products were analyzed by electrophoresis on 1.5% agarose gel (w/v) containing ethidium bromide to check their size (160 bp) and estimate their concentration.

**DGGE analysis**

Denaturing gradient gel electrophoresis (DGGE) was performed using a D-Code Universal Mutation Detection System (Bio-Rad, Hercules, CA) on 16 cm × 16 cm ×1 mm gels. Electrophoresis was performed in 8% (w/v) polyacrylamide (acrylamide:Bis 37.5:1) gels containing a urea-formamide gradient from 37.5 to 50%. A 100% denaturing solution contained 7 M urea and 40% (v/v) formamide and 7M urea. Electrophoresis was performed in 1×Tris-acetate- EDTA (TAE) buffer at a constant voltage of 120 V at 60 °C for approximately 8 h. Gels were stained with SYBR Green I (Invitrogen) diluted to 10^-4^ in 1×TAE for 30 min. Stained gels were analyzed by using the software of Quantity-One (Bio-Rad). The similarities between DGGE profiles were determined using the unweighted-pair group method with the arithmetic average (UPGMA) clustering algorithm.

**Sequencing of selected bands from DGGE gels**

Representative bands were excised from DGGE gels with a sterile scalpel, and eluted at 4 °C overnight in 20μl sterile water. A 3μl aliquot was reamplified by PCR using the original primers without the GC-clamp (2 min at 94 °C, 30 cycles consisting of 60 s at 94 °C, 30 s at 55 °C, and 45 s at 72 °C, and finally 5 min at 72 °C). PCR products that migrated as a single band and at the same position with respect to the control were amplified with the primer without the GC clamp, purified, and sent to a commercial sequencing facility (Invitrogen, USA) for sequencing. Searches in the GenBank with the BLAST program were performed to determine the closest known relatives of the partial 16S rDNA sequences obtained. Identities of isolates were determined on the basis of the highest score. Phylogenetic trees were constructed from evolutionary distances by the neighbor-joining method through the MEGA software (version 4.0).

**Transmission electron microscopy**

For transmission electron microscopy, the intestines were immersion fixed in 2.5% glutaraldehyde. After fixation, the specimens were rinsed with PBS and postfixed in 1% OsO_4_. The tissue samples were dehydrated by graded ethanol and then embedded in Epon 812. Ultrathin sections were cut with an Ultramicrotome System 2128 (Ultratome, Bromme, Germany). Subsequently, sections were collected on 200 mesh cooper grids and stained with uranyl acetate followed by lead citrate. Sections were photographed using a JEOL 1200EX transmission electron microscope (Hitachi, Tokyo, Japan) at 100 kV.

**Immunofluorescence labeling of tight junction proteins**

Immunofluorescence labeling was performed on the allograted intestine and recipients’ native ileal tissues that were embedded in OCT Compound (Sakura Finetek USA, Inc, Torrance, CA, USA). Tissues were sectioned at 5 µm thickness and fixed in cold acetone (-20°C) for 10 min. Tissue sections were blocked with 1% bovine serum in PBS for 15 min before incubation with primary antibodies. After then, sections were incubated with Rabbit anti-occludin and claudin-3 polyclonal antibodies, and mouse anti-claudin-1 and 5 monoclonal antibodies over night at 4 °C. Primary antibodies were diluted at 1:150. Sections were washed with PBS and incubated for 30 min with Alex Fluo 635-conjugated anti-mouse or anti-rabbit secondary antibodies (1:100). The sections were then analyzed using a Leica TCS SP5 confocal scanning microscope (Leica Microsystems, Heidelberg GmbH, Mannheim, Germany).

**TJ barrier function measurement**

Changes in TJ barrier function was assessed by biotin tracer experiment according to the previous report [1]. EZ-link Sulfo-NHS-Biotin was used as a molecular tracer in this study.

Reference

1. Guttman JA, Li YL, Wickham ME, et al. (2006) Attaching and effacing pathogen-induced tight junction disruption *in vivo*. Cell Microbiol 8: 634-645.
